# The acute effects of cocoa flavanols on temporal and spatial attention

**DOI:** 10.1007/s00213-018-4861-4

**Published:** 2018-03-03

**Authors:** Aytaç Karabay, Jefta D. Saija, David T. Field, Elkan G. Akyürek

**Affiliations:** 1 Department of Psychology, Experimental Psychology, University of Groningen, Grote Kruisstraat 2/1, 9712 TS Groningen, The Netherlands; 2 Department of Psychology, Centre for Integrative Neuroscience & Neurodynamics, University of Reading, Reading, UK

**Keywords:** Cocoa flavanols, Rapid serial visual presentation, Visual search, Attention, Temporal integration

## Abstract

In this study, we investigated how the acute physiological effects of cocoa flavanols might result in specific cognitive changes, in particular in temporal and spatial attention. To this end, we pre-registered and implemented a randomized, double-blind, placebo- and baseline-controlled crossover design. A sample of 48 university students participated in the study and each of them completed the experimental tasks in four conditions (baseline, placebo, low dose, and high-dose flavanol), administered in separate sessions with a 1-week washout interval. A rapid serial visual presentation task was used to test flavanol effects on temporal attention and integration, and a visual search task was similarly employed to investigate spatial attention. Results indicated that cocoa flavanols improved visual search efficiency, reflected by reduced reaction time. However, cocoa flavanols did not facilitate temporal attention nor integration, suggesting that flavanols may affect some aspects of attention, but not others. Potential underlying mechanisms are discussed.

## Introduction

Flavonoids such as flavones, flavanols, flavanones, and flavonols, which are a subclass of phenolic compounds, are found in various dietary sources. Flavanols, which are found in green tea, cocoa products, and red wine, are one of the 8000 polyphenols (Bravo 1998). The effect of flavanols on human health has drawn considerable attention, since flavanol-containing products are consumed by many people in western countries on a daily basis. In this study, we focused on cocoa flavanols due to its higher flavanol content than other flavonoid-containing products such as tea and wine (Lee et al. 2003). Long-term studies revealed that sustained intake of cocoa flavanols (CF) decreases insulin resistance and provides benefits to cardiovascular health (Hooper et al. 2012). Moreover, neuroprotective effects of CF in elderly people have been observed (Vauzour et al. 2008; Mastroiacovo et al. 2014). Various acute effects (i.e., occurring directly after consumption) on brain function have also been observed, on both physiological and cognitive measures (for a review on the cognitive effects of both acute and long-term use of cocoa flavanols, see Socci et al. 2017). In general, relative to acute physiological effects of cocoa flavanols administration (e.g., immediate cardiovascular effects), behavioral results have not been as unequivocal.

Starting with the latter, direct evidence for some (albeit limited) effects of CF consumption on cognitive functions was provided in a behavioral study conducted by Scholey et al. (2010), who found positive acute effects of CF consumption on cognitive task performance and mental fatigue. The standardized cognitive demand battery (CDB) test was used in a counterbalanced, double-blind, placebo-controlled design.^1^ Significant improvements as a result of acute CF consumption were found on the serial threes task, which involves counting backwards in threes from a random number between 800 and 999. No improvement was observed on the more difficult version of that task, the serial sevens. On a rapid visual information processing task, which required participants to monitor series of digits (at 100 digits per minute), and press a button when there are three odd digits in a row, no improvements in task accuracy due to CF were found either. However, significant improvements in reaction time were observed in their high dose CF condition (994 mg CF) in the third and fourth cycle of CDB (total of 6 cycles). Finally, mental fatigue was significantly improved after consumption of a low dose of CF (520 mg), as measured by the scores on a visual analogue scale on which participants self-rated their mental fatigue.

Similarly, Massee et al. (2015) investigated acute and sub-chronic effects of CF on cognition using the CDB, in a randomized, double-blind, placebo-controlled, parallel design study. Significant improvements were found after consumption of CF (250 mg) in the serial sevens subtraction task, but only in the first cycle of the CDB (on a total of 3 cycles). Mental fatigue was also alleviated by CF in this study. Nevertheless, the CF effects on the CDB were not entirely consistent with those of Scholey et al. (2010). Possible reasons could be methodological differences: for instance, in the amount of CF administered (250 mg as an experimental condition and 0 mg flavanol as a placebo vs. 500 mg as a low dose and 994 mg as a high dose), in the number of CDB cycles, and in the design (crossover vs. parallel).

Field et al. (2011) used dark chocolate (733 mg CF) and white chocolate (containing only a trace amount of CF) in a counterbalanced crossover design. They investigated effects of CF on visual and cognitive tasks. They found significant improvements of acute CF consumption on visual contrast sensitivity, and reaction time in motion integration, visual working memory, and choice reaction time tasks. However, since dark and white chocolate could be distinguished by participants, the study was not double-blind, and placebo effects might thus have contributed to the results. Furthermore, caffeine and theobromine were present in the dark chocolate while they were absent in the white chocolate. Hence, caffeine and theobromine levels in these two treatment conditions did not match, which could also explain the observed effects.

Grassi et al. (2016) investigated whether CF consumption counteracts effects of sleep deprivation on cognition, next to cardiovascular parameters. Participants visited the lab the night before each experimental session, and they either slept (sleep condition) or did not sleep (deprivation condition). Afterwards, participants consumed either flavanol rich (520 mg) or flavanol poor (88.5 mg) dark chocolate. Each participant visited the lab four times, so that a double-blind crossover design was realized. Ninety minutes after CF consumption, participants took psychomotor vigilance and 2-back tasks. For women, performance in the 2-back task did not decrease after sleep deprivation, when they had consumed flavanol-rich dark chocolate, while their performance did decrease when they consumed flavanol-poor dark chocolate. The study thus suggested that cocoa flavanols can restore working memory performance after sleep deprivation in women, implicating it might attenuate the effects of mental fatigue. Some caution must be exercised when interpreting these outcomes, however, because caffeine and theobromine levels were not matched between conditions (109 mg caffeine and 1200 mg theobromine in the flavanol rich condition vs. 49 mg caffeine and 419 mg theobromine in the flavanol poor condition), which may have confounded the effect.

Finally, another study of both acute and chronic effects on cognitive performance and mood did not show effects of CF on cognitive performance, but only on mood: Pase et al. (2013) tested both acute and chronic effects of CF on cognitive performance in the so-called Cognitive Drug Research computerized assessment system, which is intended to test both attentional and (working) memory functions, using a randomized, placebo-controlled, double-blind, parallel groups design. Participants took the assessment 1, 2.5, and 4 h after they had consumed a CF-containing drink (0 mg, 250 mg, 500 mg CF), as a measure of acute effects, and they were tested again after 30 days of CF consumption. Neither of these tests provided any evidence for an effect of CF on cognitive performance. Self-reported mood was not affected after acute intake of CF either. However, after 30 days of daily CF intake, self-reported calmness and contentedness scores were significantly greater than the baseline scores in the high-flavanol condition. There was no improvement of CF on mood in low flavanol and placebo condition. It must nonetheless be noted that participants had a lunch break after the first testing session in this study, which means that post-prandial factors may potentially have contributed to the negative findings, particularly with regard to acute effects.

As alluded to, these various behavioral effects should obviously be rooted in transient physiological changes induced by CF consumption. The (potentially) beneficial physiological effects of CF depend in part on its ability to activate nitric oxide (NO) synthesis in vitro (Karim et al. 2000) and vivo (Fisher et al. 2003). NO has multiple biological functions, two of which could potentially explain the reports of enhanced cognition due to CF consumption—vasodilatory effects and neurotransmission. NO systems mediate vasodilation in blood vessels, including cerebral arteries, by stimulation of guanylate cyclase (Calver et al. 1992). Consistent with this, several studies have confirmed that consumption of CF influences cerebral blood flow (Francis et al. 2006). However, because vasodilation is not the only relevant biological role of NO, it cannot be assumed that the cerebral blood flow effect of CF consumption is solely responsible for effects of CF on measures of cognitive performance. Independently of its blood flow effects, CF also influences neuronal signaling pathways (Spencer 2007). Specifically, NO acts as a neurotransmitter, although its behavior and effect is somewhat different to the classical neurotransmitters (Garthwaite 1991), and this offers an alternative explanation of the cognitive effects of CF.

To date, there is no strong evidence in favor of either mechanism. In one study, Francis et al. (2006) showed increased cerebral blood flow 2 h after consumption of a flavanol-rich cocoa drink (containing 516 mg CF), compared to a low flavanol condition (39 mg CF) in a counterbalanced, double-blind, crossover design. However, even though increased blood flow in the brain should likely result in better cognitive performance overall, Francis et al. (2006) did not find behavioral evidence that CF increased performance in their task-switching test. This null result might have occurred because participants were trained to have less than 5% error rate in the task, so that performance might have been at ceiling. Alternatively, it might be that the cognitive functions involved in task-switching are less sensitive to CF effects.

In another study with a counterbalanced, double-blind, crossover design by Lamport et al. (2015), more specific physiological effects were found. The authors observed increased arterial spin labeling perfusion in two clusters, the anterior cingulate cortex and central opercular cortex of the left parietal lobe, after 2 h of 494 mg CF consumption in healthy elderly adults. Modulation of attention, executive functions, and error detection are some of the functions of anterior cingulate cortex (for a review, see Bush et al. 2000). Furthermore, anterior cingulate cortex activation was previously found in attentional blink tasks (Marois et al. 2000), implicating temporal attention specifically. However, this remains indirect, as no behavioral task was performed in the study by Lamport et al. (2015).

Decroix et al. (2016) showed increased cerebral blood oxygenation due to CF intake by using a functional near infrared light attenuation (NIR) setup in a double-blind, randomized, crossover design. The authors collected cerebral oxygenation levels three times, at baseline, and at 90 min and 140 min after baseline. At 90 min after baseline, a Stroop task was administered to investigate whether CF influences cerebral blood oxygenation levels and executive cognitive functions. Increased cerebral oxygenation as a result of 900 mg CF intake was observed, but there was no behavioral evidence that CF improved Stroop performance. Particularly because the Stroop task lasted for only 5 min, one account for the lack of a CF effect is that the task was too short to allow modulation of executive functions, as evidence from Grassi et al. (2016) suggested mental fatigue might be mediating CF effects on these functions.

Taken together, it seems fair to conclude that the evidence for acute effects remains mixed. It is conceivable that the mixed pattern of results has arisen because results from standardized test batteries do not always specifically target individual cognitive functions (such as only attention or working memory) in isolation. Also, methodological differences across studies, including CF dosage and administration (e.g., chocolate bar or beverage), and designs (crossover vs parallel), may explain some of the mixed results. Nevertheless, from the available evidence to date, such as the physiological data (Lamport et al. 2015), and the reaction time effects in the CDB (Scholey et al. 2010), we speculate that CF might particularly affect attention. The present study sought to provide a decisive test of this possibility, firstly by employing a randomized, counterbalanced, double-blind, placebo- and baseline-controlled, crossover design, which was also pre-registered; its full specification, including analysis plan and hypotheses were published online on the Open Science Framework website (www.osf.io) in advance. Secondly, we also used a novel approach to specifically target attentional deployment in both time and space: we chose experimental tasks that are commonly used by attention researchers, rather than tasks from cognitive test batteries. Doing so allowed for a more focused examination of attention in isolation, rather than as one part of a multidimensional array of cognitive functions that participants are typically required to perform in test batteries.

To investigate whether CF influences attention in time and space specifically, a hybrid attentional blink/temporal integration task and a visual search task were implemented in the present study. The first task was a rapid serial visual presentation (RSVP) task. In a classical RSVP task, distractors and targets are successively shown on the same central location on a screen in a very short time period (~ 10 visual items per second), and the task is to identify and report target items among distractor items. Typically, two targets are inserted in the stimulus stream, and when the second target follows within 200–500 ms of the first target (Raymond et al. 1992), its identification is difficult, and this is known as the attentional blink (AB) phenomenon. Although various factors can influence second target identification accuracy in RSVP (for a review see Dux and Marois 2009), the AB is still generally regarded as closely tracking the deployment of attention across time. Furthermore, in our hybrid task, target integration, which is the perception of a combined, integrated compound target out of two successively presented targets, could be assessed separately. Integration is one way to avoid the AB (Akyürek et al. 2012; Bowman and Wyble 2007). Hence, this task can also shed light on temporal integration mechanisms that may modulate temporal attention, thereby providing a more sensitive measure of possible CF effects. Visual search (VS) constituted the second task, which is used to investigate the accuracy and efficiency of the deployment of spatial attention (for a review, see Wolfe 1998). The task is to detect whether a single target item was present or absent in a visual array consisting of a number of items. The difficulty of visual search, which is primarily reflected in reaction times to the search array, depends on the ease of discrimination of a target element amidst distractors, and the number of elements that must be inspected—except in the case where the target is very different from the distractors, in which case there is no effect of the number of elements in the visual display, commonly known as pop out search. In our task, search difficulty was manipulated by introducing a second salient item in the search arrays, which either matched or did not match the relevant target features (cf. Akyürek and Schubö 2011).

Taken together, two main questions were addressed in this study: (I) Whether acute CF consumption facilitates temporal attention and/or integration and (II) Whether acute CF consumption enhances spatial attention in terms of accuracy and/or efficiency (i.e., reaction time).

## Methods

### Participants

Forty-eight (24 female) healthy non-smoking volunteers participated in the study (mean age = 22.15 years, range = 18–29, SEM = .01). None of them were previously diagnosed with any vascular disease, with a health disorder affecting metabolism, or with neurological or psychiatric disorders. They were not following a medically restricted diet or taking vitamin supplements, they were not pregnant or breastfeeding, and they had a body weight between 55 and 90 kg. All subjects had best-corrected visual acuity of 20/20 (Snellen) at a test distance of 35 cm and were able to pass the Ishihara color vision test. The mean height of male participants was 174.5 cm (range = 160–198 cm, SEM = 1.67), and the mean height of females was 165.6 cm (range = 153–187 cm, SEM = 1.50). The mean body weight of male participants was 72.7 kg (range = 57–90 kg, SEM = 1.90), and the mean female weight was 61.8 kg (range = 55–78 kg, SEM = 1.46). Written informed consent was obtained prior to participation and participants received 25 euros remuneration. The study was approved by the ethical committee of the Psychology Department of the University of Groningen (approval number ppo-014-227) and was conducted in accordance with the Declaration of Helsinki (2008).

### General procedure

Participants visited the lab on four separate days, with a washout period of at least 1 week in-between to ensure any effects of the previous session had dissipated. Each session started at a fixed time: At 10:00, 11:00, 14:00, or 15:00. Subjects visited the lab at the same time and day of the week for each of their sessions, to avoid introducing differential diurnal effects. On the day of, and the day before each lab visit, the participants were asked to abstain from consuming products that contain caffeine, alcohol, high concentrations of flavonoids or theobromine (cf. Field et al. 2011), or herb supplements. A list with products that contain these prohibited components was given to the participants, and the importance of compliance was stressed before the first visit and at each visit. The list contained products such as coffee, tea, alcoholic beverages, and dark (high cocoa content) chocolate, as well as herbal teas and supplements (cf. Sokolov et al. 2013). Participants were asked whether they complied at each visit, while making it clear that although their data would have to be discarded in case of non-compliance, they would still receive financial reimbursement as agreed (promoting self-report honesty).

A researcher that was not otherwise involved in the administration of the experiment served the drinks containing the experimental products (or not, see below) to the participants 2 h prior to the experiment. The delay between consumption and experiment was chosen to allow proper CF uptake by the body (Francis et al. 2006; Lamport et al. 2015). Across sessions, each participant consumed all four drinks in a randomly assigned order, which was counterbalanced between subjects. Between consumption and test, participants were invited to wait in the library, and asked not to consume anything, except for one additional glass of water, if desired. Participants were not aware of the type of drinks they consumed (see below), except for the baseline condition, since its visual appearance and taste did not mask the fact that it was a mixture of water and sugar. Another researcher, who was not aware of the experimental product that the participants had consumed, handled subsequent data collection in the lab. Participants took the visual acuity test and the color blindness test at the first session, where their weight and height were also measured. Each participant was seated approximately 60 cm away from the screen in a dimly lit, sound and light attenuated testing cabin. Participants completed two experimental tasks which were counterbalanced across participants: A dual-target rapid serial visual presentation task, and a dual-singleton visual search task.

### Apparatus

The experimental tasks were presented on a 22” CRT monitor (Iiyama MA203DT) with a refresh rate of 100 Hz, at 16 bit color depth. Experimental tasks were programmed in E-prime 2.0 Professional (Psychology Software Tools) and executed under the Windows 7 operating system. Responses were collected with a standard USB keyboard.

#### Experimental product

High-flavanol Acticoa ™ cocoa powder, containing 8.3 g flavanols/100 g, as well as alkalized cocoa powder that contained no flavanols, were provided free of charge by Barry Callebaut. No other support, including sponsoring or financing of any kind was given, and the company had no other involvement in the study. Both types of cocoa powder were otherwise closely matched, including on levels of caffeine and theobromine, which both could potentially reduce fatigue and enhance alertness. The most notable difference was that the alkalized powder necessarily contained more potassium, 4790 mg K/100 g, compared to 1500 mg K/100 g for the high-flavanol powder. The cocoa was served as drink with 300 ml hot water and 20 g sugar to enhance palatability. There were four conditions: baseline, placebo, low dose, and high dose. Neither baseline nor placebo condition contained any CF. The baseline condition consisted of 20 g sugar dissolved in warm water, while the placebo condition additionally included 11 g alkalized cocoa powder. The low and high CF doses consisted of mixed cocoa powder, so that the drink contained 374 mg CF in the low-dose condition and 747 mg CF in high-dose condition. To this end, the low-dose condition included 4.5 g high-flavanol cocoa powder, and 6.5 g alkalized cocoa powder, while the high-dose condition contained 9 g high-flavanol cocoa powder and 2 g alkalized powder. Further details of the composition of the cocoa powders are listed in Table 1.

**Table 1:** Nutritional composition of the study treatments

	Baseline	Placebo	Low dose	High dose
Alkalized cocoa powder (g)	0	11	6.5	2
Flavanol (mg)	0	0	0	0
Energy (kcal)	0	33.5	19.8	6.1
Protein (mg)	0	2442	1443	444
Fat (mg)	0	1210	715	220
Caffeine (mg)	0	22	13	4
Theobromine (mg)	0	231	136.5	42
High-flavanol cocoa powder (g)	0	0	4.5	9
Flavanol (mg)	0	0	373.5	747
Energy (kcal)	0	0	15.5	31.1
Protein (mg)	0	0	1008	2016
Fat (mg)	0	0	630	1260
Caffeine (mg)	0	0	9	18
Theobromine (mg)	0	0	94.5	189
Sugar (g)	20	20	20	20
Hot water (ml)	300	300	300	300

#### Experimental tasks

##### Attentional blink/integration task (RSVP)

An RSVP composed of distractors and targets, in which temporally segregated targets could also be temporally integrated into a single percept, was shown in the center of the screen on a light gray background (RGB 192, 192, 192). On each trial, participants were to focus on the continuous stream of visual items, and identify the first target (T1) as well as the second target (T2), if present, amidst the distractors. Screen resolution was set to 1024 × 768 pixels. Distractor stimuli were chosen from the alphabet without replacement on each trial and presented in black 52 pt. bold Courier New Font. Target stimuli consisted of 1–4 black corner segments of a square (see the Appendix for a set of all possible targets). To avoid feature overlap, T2 never comprised a corner segment that was used for T1 in the same trial. Target stimuli extended to an area of 60 by 60 pixels (2.22° by 2.22° of visual angle) and were shown in the center of the screen. The width of each corner segment was 7 pixels (.26° of visual angle) and the length was 23 pixels (.85° of visual angle) so that the area of each corner segment was 273 pixels square. The gap between adjacent corner segments was 8 pixels (.3° of visual angle).

**Fig. 1:**
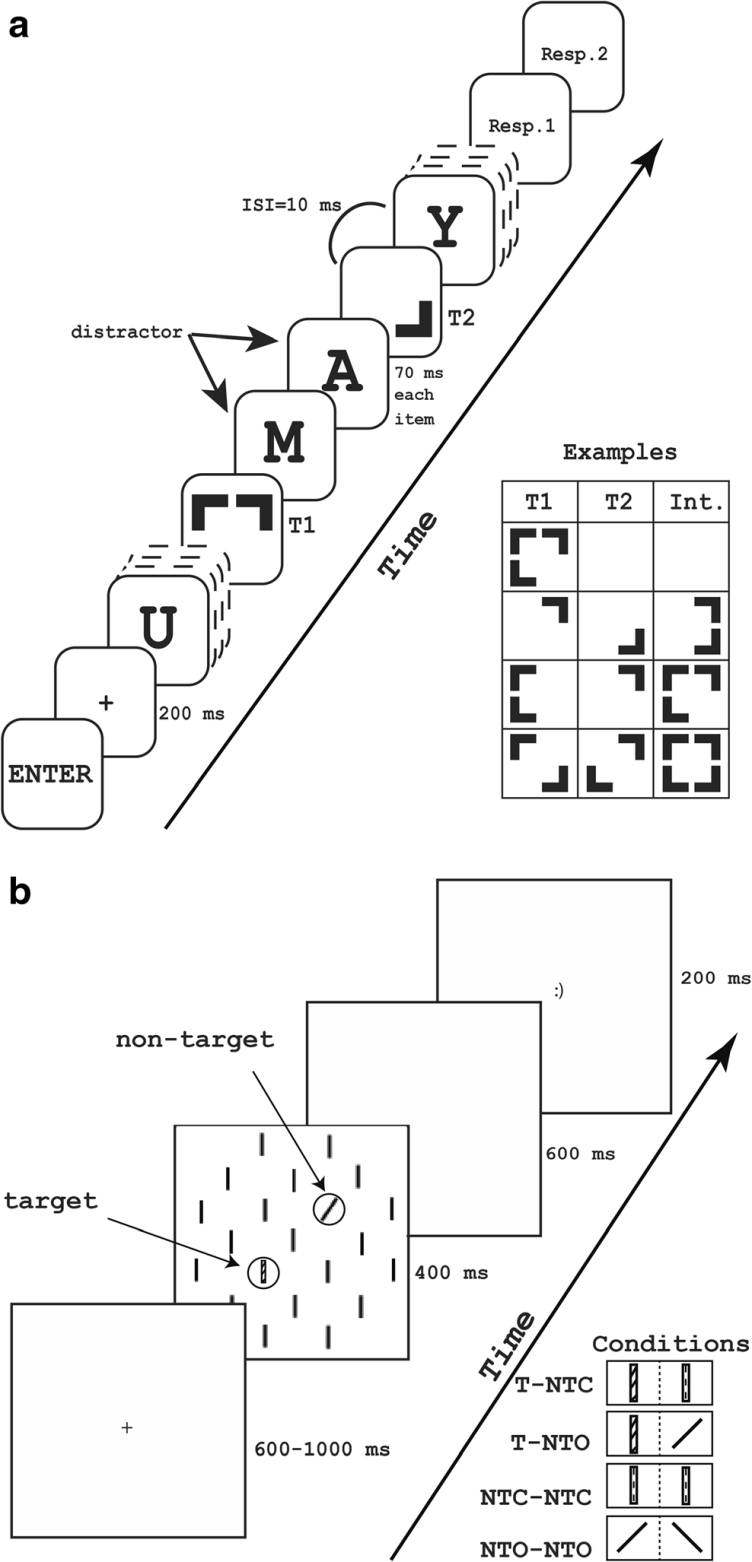
**a** An illustration of the procedure of the attentional blink/integration task at lag 3 where there are two distractors between targets. Letters are distractors, and targets appear among distractors in rapid succession. Resp. refers to response prompt. Example stimuli are shown in the box on the right bottom corner of the left panel. **b** An illustration of the procedure of the VS task. Task conditions are illustrated in the lower right corner. T indicates a (color) target, NTO refers to an orientation non-target and NTC means a color non-target. The color of non-targets was always different from the color of the target stimulus. Stimuli always consisted of solid lines. Diagonal line fills indicate color, and dashed line fills represent different colors of the non-target items

The task started with 20 practice trials, which were omitted from the analysis. Two identical experimental blocks followed, and each block consisted of 160 trials which were randomized within each block. Participants were explicitly offered a rest break between blocks. The experiment was self-paced, and participants were asked to press “ENTER” to start each trial. As shown in Fig. 1a, after 100 ms of pressing “ENTER,” a fixation cross (+) in the same font and size as the distractor stimuli appeared in the middle of the screen for 200 ms. An RSVP accommodating 18 items started after the cross. Each item was shown for 70 ms with a blank interval of 10 ms in-between. T1 was shown as the fifth or seventh item in the RSVP, which was randomized and equally distributed; 75% of the trials contained two targets while the rest only contained one target in the stream. Thus, in 25% of the trials, T2 followed T1 immediately (lag 1), as the third item (lag 3) or as the eighth item (lag 8).

At the end of each trial, two successive response prompts asked participants to enter T1 and T2. Participants were able to report the corner segments of both targets individually by pressing related keyboard buttons (1, 2, 4, and 5), followed by “ENTER.” Participants were also able to enter only one target by pressing the related keyboard buttons for one prompt and leaving the other response prompt empty by only pressing “ENTER.” Furthermore, participants were allowed to leave both response prompts empty, but were encouraged to guess at the target identities if they were merely uncertain. The total duration of the RSVP task was approximately 30 min, depending on individual response speed.

##### Visual search task (VS)

The search display was composed of 21 lines of 30 × 5 pixels (1.42° by .24° of visual angle) on a white background at a screen resolution of 800 × 600 pixels. Participants were asked to find a color-defined target stimulus within this search display. Lines were arranged in a centered circular array, and the distance between adjacent lines was 50 pixels (2.37° of visual angle). Nineteen lines in the array were oriented vertically and black, one line was always a non-target stimulus and the other remaining line was either a target stimulus or another non-target stimulus. The target stimulus was an isoluminant blue, green, or red vertical line. Non-target stimuli were either 45° clockwise/counterclockwise rotated lines or colored vertical lines that did not match the current target color. The two (non-)target stimuli were placed randomly on the search display, but one always appeared in the right visual field while the other appeared in the left visual field.

There were two experimental conditions in the task: target presence and non-target features. There were two levels of target presence, target present or target absent, and two levels of non-target features, line orientation or line color. Thus, in the target-present condition, search displays contained one (color) target, and one task-irrelevant color or orientation non-target stimulus. In the target-absent condition, there were two non-target stimuli that were either both colored or both oriented.

There were two identical blocks in the experiment and each block included three sub-blocks, whose order was randomized within subjects. The target color was different on each sub-block of 96 trials, being green, red, or blue, and the participants were instructed accordingly. Each trial started with a fixation cross with a random duration between 600 and 1000 ms. After the fixation cross, the search array appeared on the screen for 400 ms, which was followed by a blank screen for 600 ms. Participants reported whether a target stimulus was shown or not by pressing 1 (target present) or 2 (target absent) on the numeric keypad of a standard keyboard. Participants were asked to respond as fast as possible and their response time was restricted to 1000 ms in total. A happy smiley appeared for 200 ms as a feedback for correct responses, and an unhappy smiley for late and incorrect ones (Fig. 1b), after which the next trial commenced. The duration of the VS task was approximately 25 min.

### Design and analysis

As indicated, a randomized, double-blind, baseline- and placebo-controlled, counterbalanced, crossover design was used. Each participant was tested under four conditions of CF: baseline, placebo, low dose (374 mg CF), and high dose (747 mg CF), separated by a 7-day washout period. Treatment order was randomized and counterbalanced between participants to prevent any differential learning effects from influencing the results.

A 4 (CF) × 3 (lag) design was used for the AB/integration task.^2^ In order to investigate the attentional blink, the mean percentage of T2 identification in the trials in which T1 was identified correctly (T2|T1) was calculated, as is the common practice (e.g., Chun and Potter 1995). To measure temporal integration, trials in which T1 and T2 were reported as a single, integrated percept in one of the response prompts were counted, with the additional requirement that the other response prompt was left empty. Furthermore, lag-specific analyses of the effect of CF on both T2|T1 accuracy and temporal integration were planned a priori, as both attentional blink and temporal integration are known to affect specific lags only. Thus, for temporal integration, for instance, Lag 1 was focused on, since temporal integration of targets in RSVP occurs mostly there (e.g., Karabay and Akyürek 2017). Participants with an overall T1 or T2 accuracy below 25% were considered unable to perform the task well in general, and omitted from the RSVP data analysis. Four participants were consequently excluded due to low performance; one participant was excluded since his data file was empty meaning either there was a problem with response input or the task was not completed correctly. The outcomes of the experiment did not change without these exclusions.

A 4 (CF) × 2 (target presence) × 2 (non-target features) repeated measures ANOVA was used to analyze performance in the VS task for both accuracy and reaction times. Reaction times were averaged from correct trials only, and reaction times less than 100 ms were excluded from the analysis, since they were considered as a random or anticipatory response. None of the participants were excluded from the analysis of the VS task.

SPSS 23.0 was used for the repeated measures ANOVA analyses in both the AB/integration task and VS task, and Greenhouse-Geisser correction was applied when appropriate. Tukey (HSD) was used for pair-wise comparisons to characterize interaction effects. A detailed overview of all means underlying the analyses in both tasks, as well as a correlation matrix between RT and accuracy in the VS task, can be found in the Appendix.

### Preregistration and data availability

In the interest of scientific transparency the present study was fully pre-registered on the Open Science Framework with the identifier zfg85 (https://osf.io/zfg85; 10.17605/OSF.IO/ZFG85). This public pre-registration comprised the design, hypotheses, analysis approach, randomizations, and the experimental programs (including instructions). The data collected for this study, as well as the analysis scripts that were used, have since been uploaded with the identifier 2snuy (https://osf.io/2snuy; 10.17605/OSF.IO/2SNUY).

## Results and discussion

### AB task

**Fig. 2:**
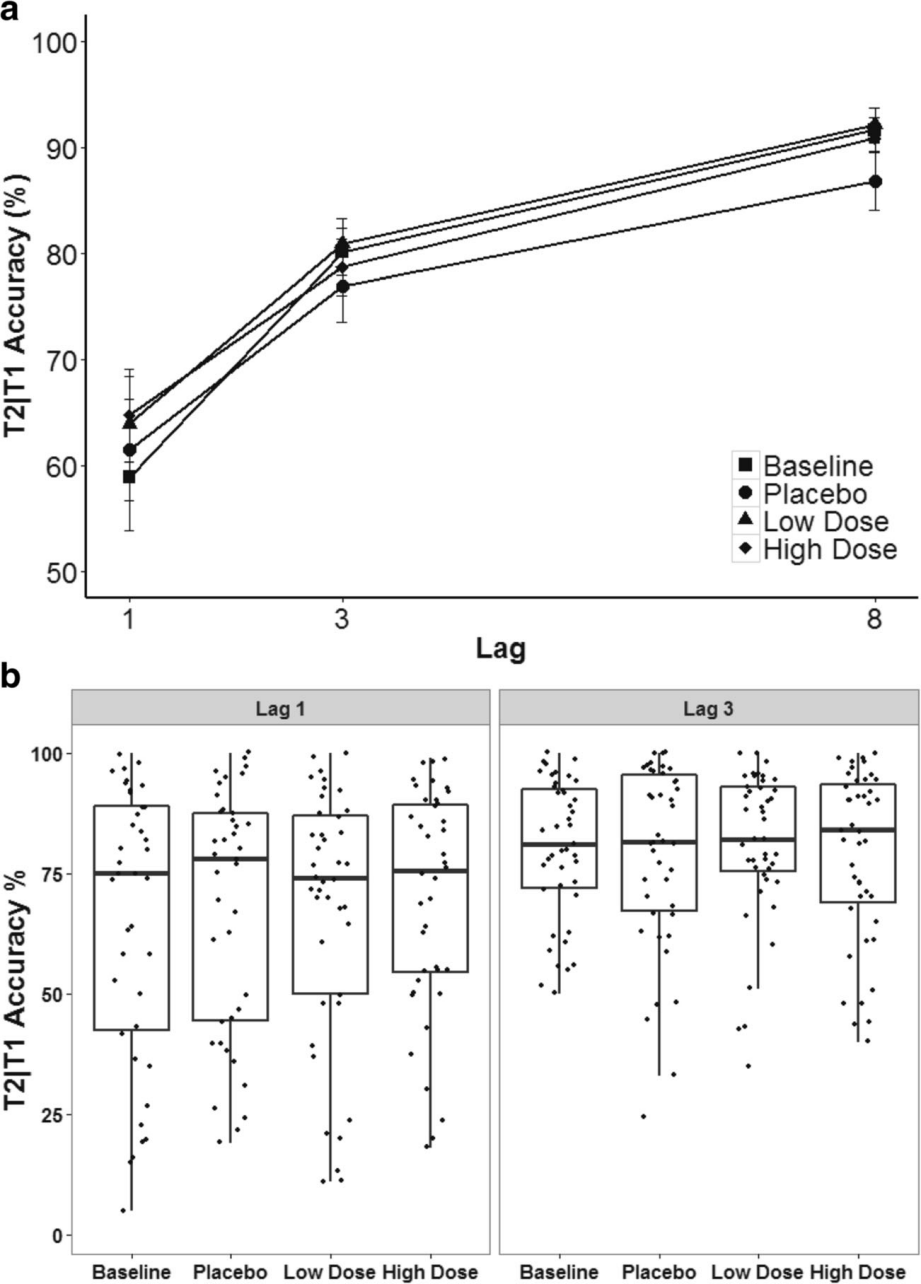
**a** Average T2|T1 (T2 accuracy given that T1 was identified correctly) in percent correct. Error bars represent ± SEM. **b** Whisker plot of lag-specific T2|T1 performance. Dots represent individual data points

T1 accuracy averaged 76.62% (SEM = 1.49%) in one-target trials in the AB task. Mean T1 accuracy across all conditions in two-target trials was 69.08% (SEM = 2.38%), and overall T2 accuracy was 64.58% (SEM = 2.64%). A significant main effect was found for lag on T2|T1 accuracy, *F*(1, 49) = 50.94, *MSE* = .12, *p* < .01, *η*^2^_*p*_ = .55. T2|T1 accuracy averaged 62.3% at Lag 1, 79.2% at Lag 3, and 90.4% at Lag 8. Neither a main effect of CF nor an interaction effect of CF and lag on T2|T1 accuracy were found, *F*(2, 90) = 1.04, *MSE* = .05, *p* = .36, *η*^2^_*p*_ = .02, and *F*(4, 160) = 2.41, *MSE* = .01, *p* = .054, *η*^2^_*p*_ = .05, respectively. Lag-specific follow-up analyses showed that the effect of CF on T2|T1 accuracy was not significant, neither at Lag 1 nor at Lag 3, *F*(3, 112) = 1.12, *MSE* = .03, *p* = .35, *η*^2^_*p*_ = .03; *F*(2, 80) = 1.03, *MSE* = .02, *p* = .36, *η*^2^_*p*_ = .02. T2|T1 accuracy, therefore, varied by lag as expected, but was independent of CF condition (Fig. 2).

**Fig. 3:**
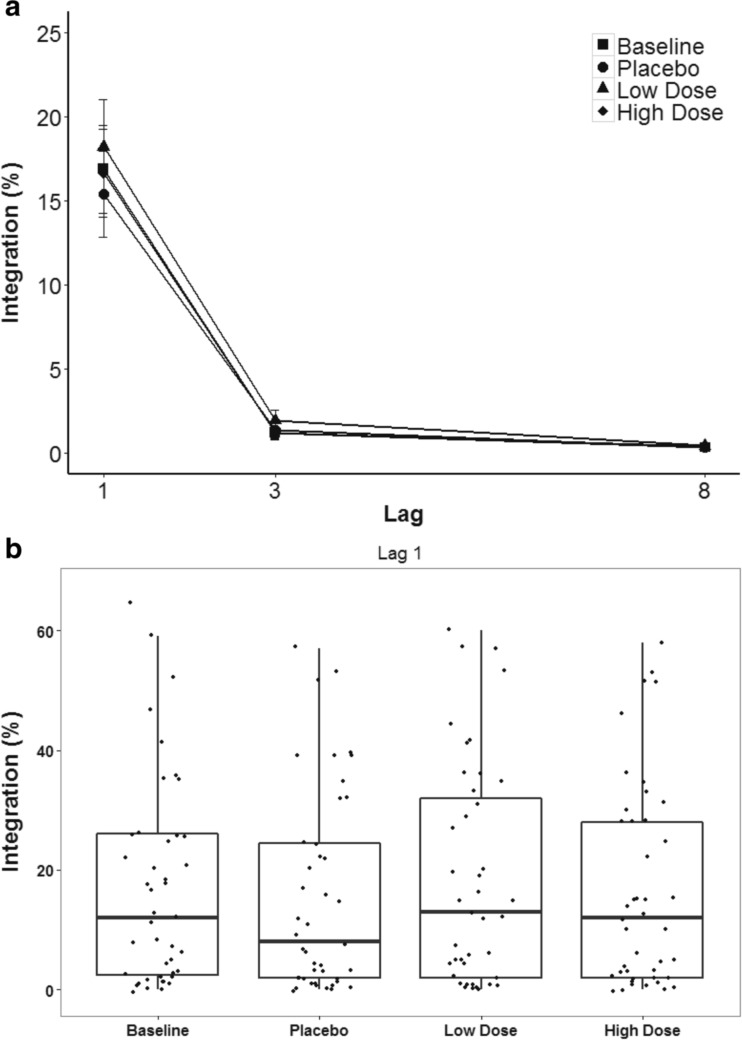
**a** Frequency of temporal integration (%) in the AB task. Error bars represent ± SEM. **b** Whisker plot of temporal integration at Lag 1. Dots represent individual data points

Lag had a significant main effect on temporal integration, *F*(1, 43) = 46.67, *MSE* = .06, *p* < .01, *η*^2^_*p*_ = .53. Temporal integration averaged 16.9% at Lag 1, 1.4% at Lag 3, and 0.4% at Lag 8, in line with the expectation that integration should only occur at the shortest lag. Both the main effect of CF and the interaction effect of CF and lag were not significant, *F*(2, 103) = 1.18, *MSE* = .00, *p* = .32, *η*^2^_*p*_ = .03, and *F*(3, 119) = .84, *MSE* = .00, *p* = .47, *η*^2^_*p*_ = .02, respectively. Further lag-specific analysis confirmed previous findings. The effect of CF on temporal integration at Lag 1 was not significant, *F*(3, 109) = .95, *MSE* = .01, *p* = .41, *η*^2^_*p*_ = .02 (Fig. 3). These analyses thus showed that although integration occurred at Lag 1 as expected, there was no evidence that CF further influenced temporal integration.

### VS task

**Fig. 4:**
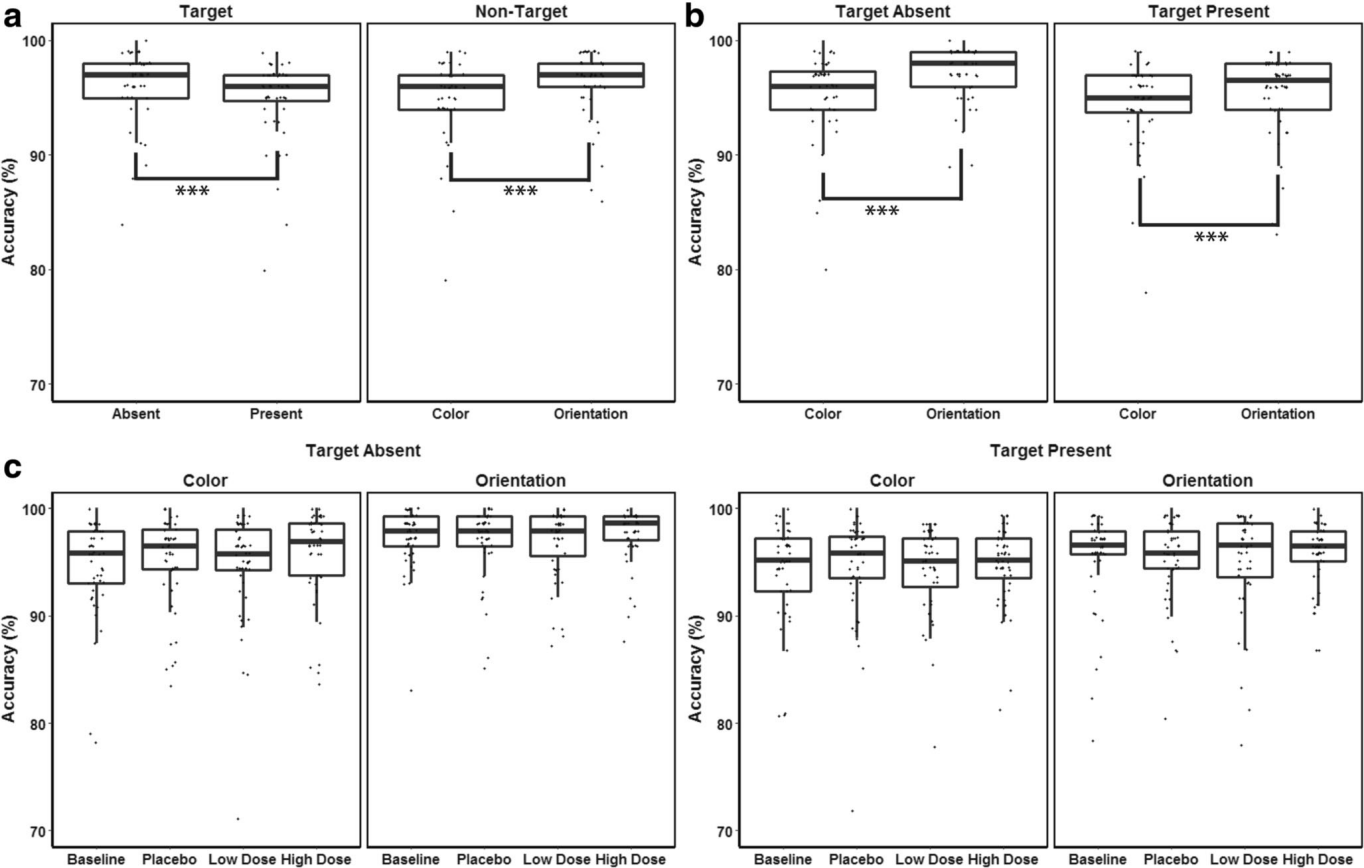
Whisker plots of the accuracy in the VS task (% correct), in which dots indicate individual data points. Significant differences are indicated with asterisks (* indicates *p* < .05; ** indicates *p* < .01, and *** indicates *p* < .001). **a** The left panel shows the main effect of target presence, the right panel shows the main effect of non-target features. **b** Interaction effect of target presence and non-target features. **c** Interaction effect of target presence, non-target features and CF

Three-way repeated measures ANOVA results revealed that accuracy in the VS task was significantly dependent on target presence, *F*(1, 47) = 27.36, *MSE* = .00, *p* < .01, *η*^2^_*p*_ = .37, as well as non-target features, *F*(1, 47) = 50.97, *MSE* = .00, *p* < .01, *η*^2^_*p*_ = .52. Mean accuracy was 94.9% in the target-present condition, and 96.1% in the target-absent condition. Task accuracy averaged 94.8% in the color non-target condition, and 96.3% in the orientation non-target condition. A significant interaction existed for target presence and non-target features, *F*(1, 47) = 8.58, *MSE* = .00, *p* = .01, *η*^2^_*p*_ = .15. Further post hoc analysis showed that accuracy in the target-absent condition, orientation non-targets resulted in significantly higher accuracy than color non-targets [*t* = 2.9, *p* < .05]. In the target-absent condition, accuracy in the orientation non-targets condition was also reliably higher than in both non-target conditions of the target-present condition [*t*_1_ = 4.3, *t*_2_ = 3.2, *p* < .01]. There was no evidence that CF, *F*(3, 125) = 1.69, *MSE* = .00, *p* = .18, *η*^2^_*p*_ = .04; the interaction of CF and target presence, *F*(3, 119) = .29, *MSE* = .00, *p* = .80, *η*^2^_*p*_ = .01; the interaction of CF and non-target features, *F*(3, 123) = 1.09, *MSE* = .00, *p* = .35, *η*^2^_*p*_ = .02; or the three-way interaction of CF, target presence and non-target features, *F*(3, 133) = .38, *MSE* = .00, *p* = .76, *η*^2^_*p*_ = .01, had a significant influence on VS accuracy. Accuracy in the VS task was thus dependent on the stimulus manipulations, but independent of the CF conditions (Fig. 4).

**Fig. 5:**
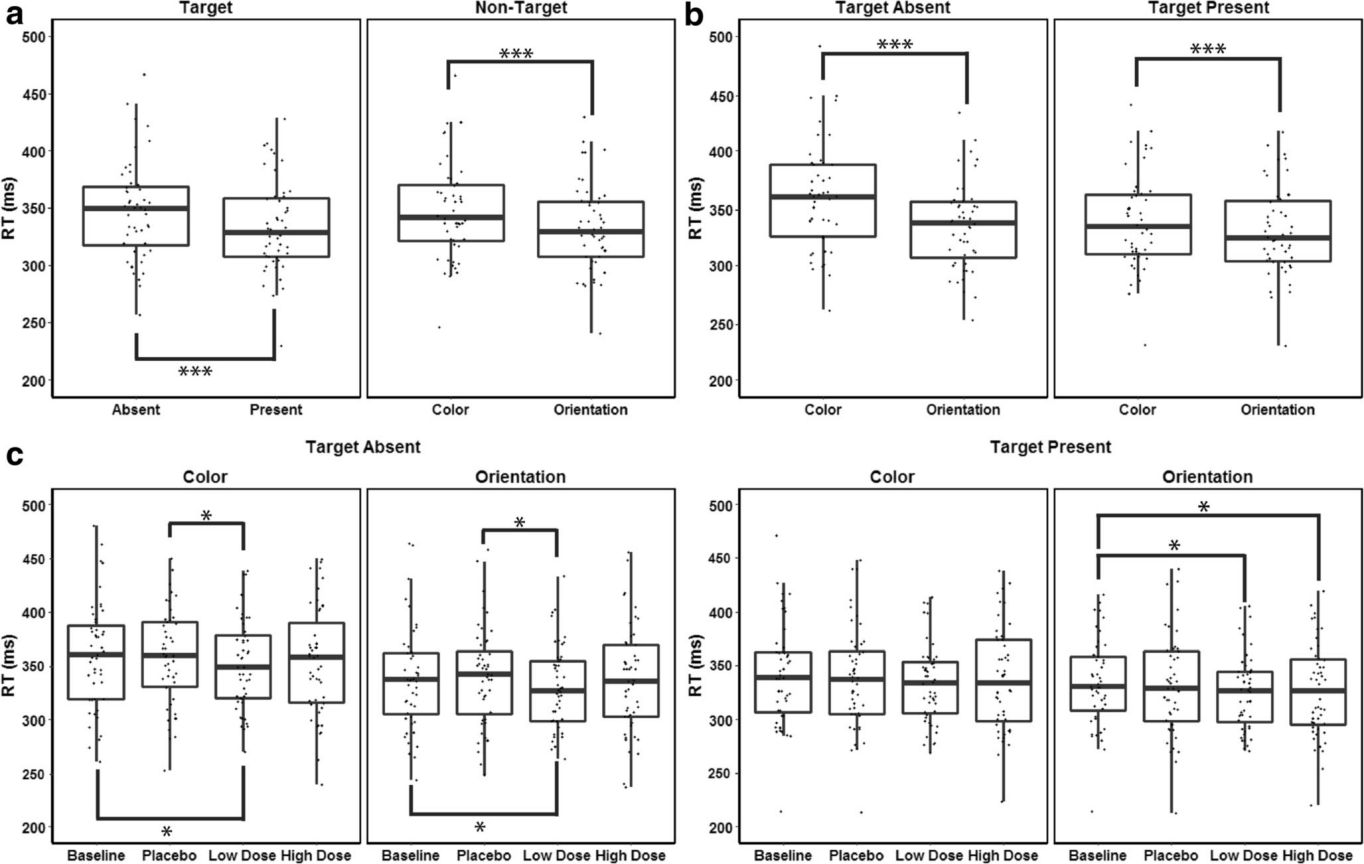
Whisker plots of RT (ms) in the VS task, where dots indicate individual data points. Significant differences are indicated with asterisks (* indicates *p* < .05; ** indicates *p* < .01, and *** indicates *p* < .001). **a** The left panel shows the main effect of target presence, the right panel shows the main effect of non-target features. **b** Interaction effect of target presence and non-target features. **c** Interaction effect of target presence, non-target features and CF

The ANOVA on reaction times in the VS task showed significant main effects of target presence, *F*(1, 47) = 21.09, *MSE* = 1752.92, *p* < .01, *η*^2^_*p*_ = .31, and non-target features, *F*(1, 47) = 176.94, *MSE* = 256.14, *p* < .01, *η*^2^_*p*_ = .79. The interaction of target presence and non-target features was also reliable, *F*(1, 47) = 80.88, *MSE* = 138.27, *p* < .01, *η*^2^_*p*_ = .63. Mean RT was 334 ms in the target-present condition and 348 ms in the target-absent condition. RT averaged 348 ms in the color non-target condition, and 333 ms in the orientation condition (see Fig. 5). Pair-wise comparisons of the interaction effect of target presence and non-target features showed that RT in the orientation non-target trials in the target-present condition was lower than in the color non-target trials in both target-present [*t* = 2.6, *p* < .05] and absent conditions [*t* = 10.2, *p* < .01]. Furthermore, RT in the color non-target trials in the target-absent condition was significantly higher than in the orientation non-target trials of the same condition [*t* = 7.8, *p* < .01], as well as higher than in the color non-target trials of the target-present condition [*t* = 7.3, *p* < .01]. The shortest RT was observed in the orientation non-target trials in the target-present condition, and the longest RT was observed in the color non-target trials in the target-present condition.

The main effect of CF on RT, *F*(3, 127) *=* 2.09*, MSE =* 1965.02*, p =* .11*, η*^2^_*p*_ *=* .04, its interaction with target presence, *F*(3, 139) *=* 1.32*, MSE =* 206.06*, p = .*27*, η*^2^_*p*_ *=* .03, as well as its interaction with non-target features, *F*(3, 133) *=* .02*, MSE =* 54.41*, p =* .99*, η*^2^_*p*_ *=* .00, were all insignificant. Crucially, a significant interaction effect of CF, target presence and non-target features, was found, *F*(3, 134) *=* 4.35*, MSE =* 45.63*, p* < .01*, η*^2^_*p*_ *= .*09. RTs in the low CF dose condition in both the color and orientation non-target trials of the target-absent condition were significantly lower than in the baseline and placebo CF condition [*t*_1_ = 2.2, *t*_2_ = 2.3, *t*_3_ = 2.0, *t*_4_ = 2.0, *p* < .05, respectively]. Moreover, RTs in the low and high CF dose condition in the orientation non-target trials in the target-present condition were lower than in the baseline condition [*t*_1_ = 2.45, *t*_2_ = 2.85, *p* < .05] (see Fig. 5).

## General discussion

We investigated acute CF effects on temporal and spatial attention in young adults with a double-blind, randomized, counterbalanced, placebo- and baseline-controlled, crossover design. Our study revealed two main outcomes, namely that CF does not influence temporal attention, but that CF does decrease RT in visual search with medium effect size, and without losing accuracy, suggesting that search efficiency was improved. Faster reaction times were observed in the low dose CF condition than in the baseline and placebo conditions when the target was absent from the search array. A similar effect was observed in the low- and high-dose conditions, compared to the baseline condition, when the target was present and the non-target was a tilted line (i.e., not defined in the task-relevant feature dimension).

Even though anterior cingulate cortex activation was previously found in attentional blink tasks (Marois et al. 2000), and in which increased arterial spin labeling perfusion in resting state after CF consumption was observed (Lamport et al. 2015), the present study produced no evidence that T2|T1 accuracy or temporal integration were affected by CF. Since the anterior cingulate cortex has other functions, apart from attentional control, such as executive functions and error detection, it is possible that these cognitive functions rather than temporal attention may be more affected by CF consumption. The present findings are also compatible with previous measures of sustained attention, as found in the cognitive drug research task battery (Pase et al. 2013). Similarly, insofar as the Stroop task can be taken to reflect selective attention, previous research has not found evidence for CF effects therein either (Massee et al. 2015; Decroix et al. 2016). It must be noted, however, that factors other than attention may underlie the Stroop effect (MacLeod 1991).

CF consumption also did not facilitate VS accuracy. A ceiling effect may have occurred, because mean accuracy was above 94% in all conditions, so that there may have been no room left for CF to enhance VS accuracy. Such performance is not atypical in spatial attention tasks, and for that reason, reaction time is typically regarded as a more sensitive and indicative measure of performance than accuracy. Critically, in the current task, RT was clearly influenced by CF consumption, suggesting that the efficiency of spatial attention was improved. It was found that both low and high doses of CF consumption resulted in shorter RTs than observed in the placebo and/or baseline conditions. It is important to note here that this result cannot be attributed to a general, possibly non-cognitive speeding of responses, or retinal effects (e.g., improved contrast sensitivity): The effect of CF consumption was only expressed through an interaction with both the variables that affected search difficulty (target presence and non-target features), pointing toward a cognitive locus. Specifically, faster RTs were observed when the target was absent in the visual array, regardless of non-target features. Furthermore, when the target was present and non-target items did not share the same feature type (color vs orientation), faster RTs were observed after CF consumption.

Previous behavioral studies have also found reaction time effects of CF on various tasks, such as rapid visual information processing tasks (Scholey et al. 2010), motion integration time threshold, and choice reaction time tasks (Field et al. 2011). At the same time, and similar to the present study, rapid serial visual information accuracy was not affected by CF consumption in these studies. The current outcomes with regard to RT speeding in visual search suggest that improved efficiency of spatial attention may (in part) have driven such previously observed effects.

Alternatively, another possible explanation for the observed difference between temporal and spatial attention might be based on differences between the RSVP and VS tasks themselves, in particular the possibility to make saccadic eye movements. Stimuli were shown in the center of screen in sequential order in the RSVP task, minimizing saccadic eye movements and eye blinks (Benedetto et al. 2015). In the VS task, the target stimulus was shown on either the right or left side of the screen for 400 ms, allowing two or three saccadic eye movements on each trial in the VS task. Therefore, acute CF effects in the visual search task may also have been facilitated faster saccades. If CF influences saccadic eye movements, such effects should be seen in other tasks allowing saccades. Significant acute CF effects on tasks allowing saccades were observed in one visual spatial working memory task (Field et al. 2011). However, another study that also used a spatial working memory task (Massee et al. 2015) showed no effect of CF. It has to be noted that fundamental differences in research designs of these two studies—within subject vs between subject design, 773 mg CF vs 250 mg CF—may hinder the comparison. Since the current study did not directly measure saccadic eye movements, and in view of the mixed evidence to date with regard to a possible role for eye movements, this remains an open question for now.

### Physiological mechanisms

Although the current study was strictly behavioral, its outcomes may also shed some light on possible underlying physiological mechanisms. From previous work, it is known that acute effects of CF may be caused by two effects related to NO synthesis: on vasodilation (blood flow) and neurotransmission (Calver et al. 1992; Garthwaite 1991; Spencer 2007). In general, if vasodilation and blood flow changes in the brain are the causal factor, then beneficial effects of CF should not depend on the type of cognitive process measured, although they might occur selectively in lengthy and fatiguing testing situations where supplies of glucose and other metabolites carried in the blood could become a determinant of performance. The same argument applies to one of the other proposed mechanisms by which flavonoids might improve cognitive function—improved blood glucose regulation (Bell et al. 2015). On the other hand, if an influence on neuronal signaling via NO or some other route is the cause then CF could influence information processing itself. This hypothesis predicts an alteration in the balance between different neural and cognitive processes underlying cognition; in terms of cognitive tests, the effects of CF should be selective rather than general. Because the current outcomes show that CF has specific rather than general cognitive effects, it seems more likely that they are due to acute changes in neurotransmission, rather than in blood flow.

## Conclusion

The outcomes of the present study suggest that in a sample of young, healthy adults, the acute effects of CF consumption do not include modulation of temporal attention, but also that CF consumption does enhance the efficiency of spatial attention. There is thus evidence to conclude that although CF consumption may not generally enhance cognitive processes (cf. Pase et al. 2013), it can produce facilitation of specific cognitive functions.

## Figures and Tables

**Table 2:**
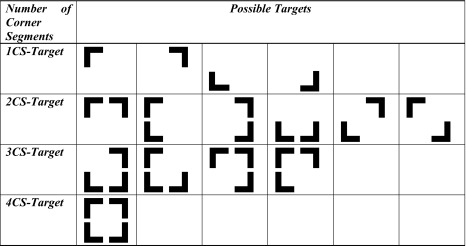
Possible targets in RSVP. CS means corner segments, and the number in front indicates the number of corner segments that the targets consisted of

## Appendix

### Possible targets in RSVP

Possible targets in the RSVP task consisted of corner segments of a square, listed in the table below. Targets were randomly chosen (without replacement) from this set, with the only requirement that the two targets did not overlap within a single trial. In other words, the targets never contained the same corner segments.

### Correlation matrix of accuracy and RT of VS task

A correlation matrix of accuracy and RT in the VS task is shown in the table below. As can be seen from the table, there were only two significant correlations between RT and accuracy. The overall pattern of correlations does not support the idea that speed might have been traded for accuracy.

**Table 3:** The Pearson correlation matrix of accuracy and RT in the VS task

	Baseline_TP_NTC	Placebo_TP_NTC	LowDose_TP_NTC	HighDose_TP_NTC
	Accuracy	Accuracy	Accuracy	Accuracy
RT	−.10	−.24	−.06	−.10
	Baseline_TP_NTO	Placebo_TP_NTO	LowDose_TP_NTO	HighDose_TP_NTO
	Accuracy	Accuracy	Accuracy	Accuracy
RT	.11	−.30*	−.07	−.50
	Baseline_TA_NTC	Placebo_TA_NTC	LowDose_TA_NTC	HighDose_TA_NTC
	Accuracy	Accuracy	Accuracy	Accuracy
RT	−.23	−.34*	−.21	−.14
	Baseline_TA_NTO	Placebo_TA_NTO	LowDose_TA_NTO	HighDose_TA_NTO
	Accuracy	Accuracy	Accuracy	Accuracy
RT	−.16	−.16	−.11	−.11

TA is target absent condition and TP represents target-present condition. NTC indicates the color non-target condition, NTO is the orientation non-target condition. Asterisks (*) indicate significant Pearson correlations (*p* < .05)

### Means and SEM in the RSVP task

**Table 4:** Overall and lag-specific means of T1 identification accuracy, T2 identification accuracy, T2|T1 accuracy and temporal integration frequency in the attentional blink task

	Baseline		Placebo		Low Dose		High Dose	
	Mean	SEM	Mean	SEM	Mean	SEM	Mean	SEM
Overall T1 ACC	70.0	2.4	67.4	3.1	71.0	2.2	67.9	2.9
T1 ACC-Lag1	41.4	3.6	41.0	4.1	41.3	3.3	41.2	3.8
T1 ACC-Lag3	82.7	2.3	78.2	3.3	83.8	2.3	79.4	2.9
T1 ACC-Lag8	86.0	2.0	83.0	3.0	87.8	1.9	83.1	2.7
Overall T2 ACC	65.2	2.6	62.8	3.5	66.2	2.6	63.4	3.2
T2 ACC-Lag1	35.8	3.9	36.6	4.5	36.0	3.9	37.3	4.1
T2 ACC-Lag3	73.2	2.8	69.2	3.9	74.3	3.0	70.1	3.6
T2 ACC-Lag8	86.7	1.9	82.6	3.1	88.2	2.1	82.8	2.8
Overall T2|T1 ACC	76.9	2.5	75.1	3.3	79.0	2.5	78.1	2.5
T2|T1 ACCLag1	58.9	5.1	61.5	4.8	63.9	4.5	64.7	4.4
T2|T1 ACC-Lag3	80.1	2.2	77.0	3.4	80.9	2.4	78.7	2.7
T2|T1 ACC-Lag8	91.7	1.2	86.9	2.8	92.2	2.7	90.9	1.4
Overall integration	6.1	1.0	5.7	1.0	6.9	1.1	6.1	1.0
Integration-Lag1	16.9	2.6	15.4	2.6	18.2	2.8	16.6	2.6
Integration-Lag3	1.2	.3	1.3	.4	1.9	.6	1.2	.4
Integration-Lag8	0.3	.1	.3	.1	.4	.2	.4	.2

ACC means accuracy and SEM means standard error of the mean. All means and SEM are presented in percentage values

### Mean accuracy, RT, and SEM in the VS task

**Table 5:** Overall and condition-specific mean accuracies and RT in visual search task

	Baseline		Placebo		Low Dose		High Dose	
	Mean	SEM	Mean	SEM	Mean	SEM	Mean	SEM
Overall ACC	95.4	.5	95.5	.5	95.2	.6	95.9	.4
TA ACC	96.0	.5	96.1	.5	95.8	.6	96.7	.5
TP ACC	94.8	.6	94.9	.6	94.7	.6	95.2	.5
NTC ACC	94.5	.6	94.9	.6	94.5	.6	95.2	.5
NTO ACC	96.3	.5	96.1	.5	95.9	.5	96.6	.4
TA and NTC ACC	94.8	.7	95.2	.6	94.7	.7	95.8	.6
TA and NTO ACC	97.3	.4	97.0	.5	96.9	.5	97.6	.4
TP and NTC ACC	94.2	.7	94.6	.7	94.3	.6	94.6	.5
TP and NTO ACC	95.4	.6	95.2	.6	95.0	.7	95.7	.4
Overall RT	344.5	6.7	343.6	6.7	334.9	5.2	339.8	6.9
TA RT	350.7	7.3	351.9	7.1	340.6	5.8	347.1	7.5
TP RT	338.2	6.6	335.2	6.8	329.1	5.2	332.1	6.7
NTC RT	352.2	7.2	351.1	7.1	342.5	5.5	347.3	7.3
NTO RT	336.7	6.3	336.0	6.4	327.2	5.0	331.9	6.5
TA and NTC RT	363.1	7.9	363.9	7.7	351.9	6.1	357.3	7.9
TA and NTO RT	338.3	6.9	339.9	6.6	329.3	5.7	336.8	7.2
TP and NTC RT	341.3	6.9	338.4	6.9	333.2	5.3	337.3	7.0
TP and NTO RT	335.2	6.3	332.1	6.7	325.1	5.1	326.8	6.3

ACC indicates accuracy, TA is target absent condition, and TP represents target-present condition. NTC indicates the color non-target condition, NTO is the orientation non-target condition. SEM means standard error of the mean. Accuracies are reported as percentage values, and RTs are given in milliseconds

## Footnotes

1 Design features such as these are commonly used to highlight specific aspects of a study. The following features are referred to in the present study: “Cross-over” means that each participant receives each experimental treatment (at different times); “parallel” is its opposite, so that different groups of participants are exposed to different treatments; “randomized” means that participants are randomly assigned to experimental groups; “counterbalanced” means that the order of tasks is not fixed but varied and distributed equally between participants; “double-blind” means that neither participant nor researcher knows the experimental treatment that is consumed by participants; and “placebo-controlled” means that an experimental treatment was used in which no substance with a therapeutic effect was present, but which was otherwise not noticeably different from the other treatments (a baseline condition is similar, apart from it being recognizable as such).

2 Originally, biological sex was included as a factor in all analyses, as it has been observed to influence effects of flavanols on human cognition (Grassi et al. 2016). However, no interaction effect of biological sex and flavanols on the RSVP and VS tasks was presently observed (*F*’s < 1.17, and *F*’s < 1.75 for the respective tasks). To simplify the report of the results, biological sex was thus omitted from the analyses throughout.
